# Randomized double-blind placebo-controlled PK/PD study on the effects of a single intravenous dose of the anti-hepcidin Spiegelmer NOX-H94 on serum iron during experimental human endotoxemia

**DOI:** 10.1186/cc12290

**Published:** 2013-03-19

**Authors:** L Van Eijk, DW Swinkels, A John, F Schwoebel, F Fliegert, L Summo, S Vauleon, JM Laarakkers, K Riecke, P Pickkers

**Affiliations:** 1 Radboud University Nijmegen Medical Centre, Nijmegen, the Netherlands; 2 NOXXON Pharma AG, Berlin, Germany

## Introduction

Anemia is very frequently encountered on the ICU. Increased hepcidin production is one of the cornerstones of the pathophysiology of anemia of inflammation. The first-in-class hepcidin antagonist NOX-H94, a PEGylated anti-hepcidin L-RNA oligonucleotide, is in development for targeted treatment of anemia of inflammation. We investigated whether NOX-H94 prevents the inflammation-induced serum iron decrease during experimental human endotoxemia.

## Methods

A randomized, double-blind, placebo-controlled trial in 24 healthy young men. At T = 0 hours, 2 ng/kg *E. coli *endotoxin was administered intravenously (i.v.), followed by 1.2 mg/kg NOX-H94 or placebo i.v. at T = 0.5 hours. Blood was drawn serially after endotoxin administration for measurements of inflammatory parameters, cytokines, NOX-H94 pharmacokinetics, total hepcidin-25, and iron parameters. The difference of serum iron change from baseline at T = 9 hours was defined as the primary endpoint.

## Results

Endotoxin administration led to flu-like symptoms. Inflammatory parameters (CRP, body temperature, leucocytes, and plasma levels of TNFα, IL-6, IL-10, and IL-1RA) peaked markedly and similarly in both treatment groups. NOX-H94 was well tolerated. Plasma concentrations peaked at 0.7 ± 0.4 hours after the start of administration, after which they declined according to a two-compartment model, with a T_1/2 _of 22.5 ± 4.28 hours. In the placebo group, serum iron increased from 19.0 ± 7.6 μg/l at baseline to a peak at T = 3 hours, returned close to baseline at T = 6 hours and decreased under the baseline concentration at T = 9 hours, reaching its lowest point at T = 12 hours. In the NOX-H94 group, serum iron concentrations rose until T = 6 hours and then slowly declined until T = 24 hours. From 6 to 12 hours post LPS, the serum iron concentrations in NOX-H94-treated subjects were significantly higher than in placebo-treated subjects (*P <*0.0001, ANCOVA).

## Conclusion

Experimental human endotoxemia induces a robust inflammatory response and a subsequent decrease in serum iron. Treatment with NOX-H94 had no effect on innate immunity, but effectively prevented the inflammation-induced drop in serum iron concentrations. These findings demonstrate the clinical potential of the anti-hepcidin drug NOX-H94 for further development to treat patients with anemia of inflammation.

